# Career histories as determinants of gendered retirement timing in the Danish and Swedish pension systems

**DOI:** 10.1007/s10433-017-0424-5

**Published:** 2017-04-17

**Authors:** Stefanie König

**Affiliations:** Department of Psychology, University of Gothenburg, Gothenburg, Sweden

**Keywords:** Retirement timing, Gender, Compensation hypothesis, Status maintenance hypothesis, Labour market attachment

## Abstract

After reforms in pension systems had taken place in most European countries within the last two decades, the concern was raised that women may be disadvantaged by these reforms. It is suggested that they are faced with a higher financial need to work longer. Retrospective data from SHARELIFE are used to run an event history analysis on the timing of the final employment exit, separately for gender, country and exit cohort. This study aims to disentangle the influence of gendered labour markets and pension regulations on retirement timing by investigating conditions in Denmark and Sweden. Some evidence was found that women compensate for lower labour market attachment due to long part-time periods by working longer, especially in younger cohorts. This seems to depend on the pension system. In countries with broad basic pensions, high replacement rates for low-income groups and fewer penalties for early retirement, the compensation is suggested to be less frequent. This study indicates the growing importance of the “compensation hypothesis” compared to the “status maintenance hypothesis” of previous careers in relation with retirement timing.

## Introduction

The potential disadvantage to women incurred by the pension system reforms around the late 1990s across European countries has been a matter of concern (Guardiancich 2012; Hofäcker et al. 2016). Individualization of pension benefits, switching from defined benefit to defined contribution, and accumulation of benefits over the lifespan are aimed at pension system sustainability. These developments shift economic risks towards individuals linked with lower labour market attachment and shorter working lives (Ebbinghaus and Neugschwender 2011; Brugiavini and Peracchi 2005). Women’s careers are commonly characterized by interruptions, part-time employment and lower income which are often not considered in the active ageing debate (Ginn 2003; Foster 2011). Pension systems seem maladaptive to more flexible careers and rising atypical employment, which are both more common among women (Hinrichs and Jessoula 2012).

Countries where women’s careers had been relatively long allow the capture of a sufficient number of late life transitions. The longest careers among women can be found in the Nordic countries (Lyberaki et al. 2010). In the present study, Denmark and Sweden are compared using the notion of the most similar case design. Both Scandinavian countries fall into the same category regarding gendered labour markets (Sainsbury 1996) and welfare regimes (Esping-Andersen 1990). They have their roots in the Beveridgean public pension system, yet differ in the undertaken reforms. This research design realizes the call for future research with respect to country-specific gender differences in career histories in relation with outcomes in later life (Möhring 2015).

By linking previous career experiences to retirement timing, a bridge between gender inequality throughout the life course and gender differences in retirement timing in a specific institutional context is constructed. Potential outcomes for the life after retirement are discussed in the conclusion.

## Context and historical development of retirement timing

**Fig. 1:**
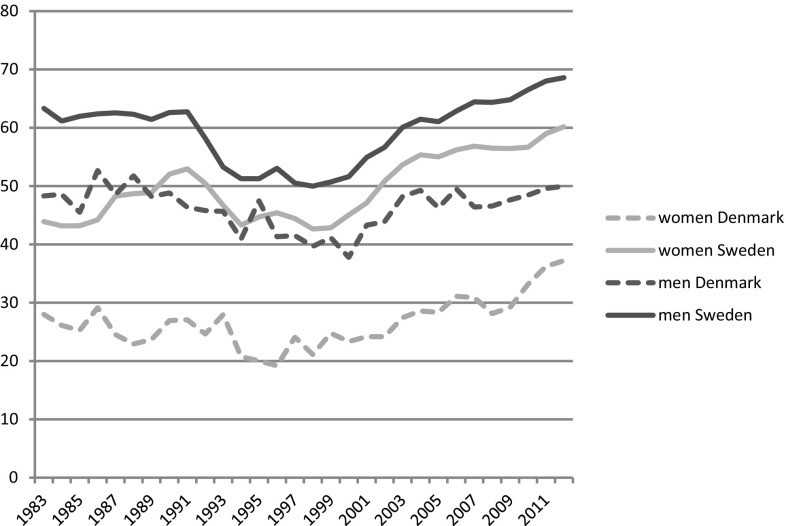
Employment rates age 60–64 by gender

Throughout the last decades, retirement timing and its context changed markedly. In times of high unemployment, older workers were frequently pushed into early retirement to rejuvenate the workforce (Buchholz et al. 2006; Ebbinghaus 2006) and to keep it competitive and adaptive to economic changes (Buchholz et al. 2011). Due to population ageing and the increasing financial burden of pensions, however, a reverse trend can be observed since the beginning of 2000 (Ebbinghaus and Hofäcker 2013). That is, the recent aim of maintaining older workers in employment has prompted the closing of early retirement pathways. But, after various reforms, early retirement is now associated with comparatively high pension income losses. Thus, public pension systems are currently less protective regarding income inequalities and employment risks in old age, than they once were (Fig. 1).

Looking at employment rates of 60- to 64-year-old workers by gender, the increase after the turn of the millennium is visible in both countries. A persistent gender gap in employment rates can be detected, especially in Denmark. Thus, women are less likely to be employed at this age since they retired or “dropped out” earlier.

## Labour market attachment and gender aspects of retirement timing

The formula for pension benefits is often constructed on typical lifelong fulltime careers. However, atypical employment, career interruptions and phases of unemployment or part-time work are increasing and common among women. Following theories of “cumulative stratification”, gender inequality throughout the life course affects financial resources and retirement decisions (e.g. O’Rand and Henretta 1999; Raymo et al. 2010; Finch 2013). Several studies related family aspects and career interruptions to retirement timing. However, the direction of this relationship is not consistent. One reason for this inconsistency may be the role of pension income. While some features of the employment history (e.g. interruptions, part-time phases) lead to lower income and thereby the need to work longer to achieve a decent pension income, the same features may lead to earlier retirement due to lower labour market attachment. Therefore, two different notions have been postulated and will be described in the next sections.

### The status maintenance hypothesis

Long and constant work careers may lead to higher retirement age, according to the “status maintenance” argument (Hardy 1991). Some studies found affirmation of this viewpoint whereby women with more stable and longer labour market participation and fewer or shorter interruptions (Pienta et al. 1994; Finch 2013), as well as women who remain employed during their childbearing years (Henretta et al. 1993; Pienta 1999), are more likely to work longer. This is explained by higher work orientation and more rewarding careers (Finch 2013). A French study found that women who had low attachment to the labour market with long periods of non-employment more often exit the labour market before the age of 60 years (Collet et al. 2013).

Marital status and children are often used as a proxy for lower labour market attachment. A study by Hank and Korbmacher (2010) found a higher likelihood for mothers and married women to retire early compared to their childless or unmarried counterparts. Comparing fathers to childless men, on the other hand, this relation is the reverse. This finding is interpreted with a weaker labour market attachment for mothers and with higher breadwinner responsibilities for fathers.

#### **H1**

Following the status maintenance hypothesis, low labour market attachment among women leads to earlier labour market exits.

### The compensation hypothesis

Contrary to the status maintenance hypothesis, an economical argumentation can be found in the literature. It is argued that certain career characteristics—often reflecting family responsibilities—lead to later retirement ages since preferred work positions and status may be achieved later (Raymo et al. 2010) and since they decrease pension income (Bardasi and Jenkins 2002; Evandrou and Glaser 2003, 2004). Career interruptions (Raymo et al. 2010; Yabiku 2000; Pienta 1999), part-time work (Lanninger and Sundstroem 2014) and atypical employment (Hinrichs 2012) were found to decrease pension income. These considerations may increase the risk of necessity-driven late retirement. Since these characteristics are more common among women’s careers, due to care responsibilities, the compensation hypothesis may be more relevant for women to compensate for accumulated “opportunity costs” (Pienta et al. 1994). Following this notion, career histories were found to have a higher explanatory power for women’s pension income compared to men’s (Möhring 2015). However, evidence for this hypothesis regarding retirement timing is scarce. A study on West-German women directly investigated the effect of years in the labour market on the retirement timing and found that longer careers up to age 50 years induced earlier transitions to retirement (Hank 2004). A Swiss study found a link between later retirement and family responsibilities, long-term interruptions and part-time periods (Madero-Cabib et al. 2016). The study by Finch (2013) from the UK found that the duration of employment in combination with working part-time affects extending working lives. If women worked longer than 25% of their careers in part-time positions, they were more likely to extend work beyond state pension age. In this study, even men were more likely to prolong their work life if they had been working part-time.

#### **H2a**

Following the compensation hypothesis, low labour market attachment leads to later exits, especially among women.

Different pension systems have the potential to counteract accumulated losses. In the following section, the most important features and changes of the pension systems shall be discussed with regard to their relevance for the compensation hypothesis. Both pension systems developed from the Beveridge model with basic flat-rate pensions and consist today of three pillars—the state pension, the occupational pensions and private pensions (Sjögren Lindquist and Wadensjö 2011; Andersen 2011). However, they differ in several aspects related to replacement rates, basic pensions, early retirement options and actuarial concepts. All in all, the Danish pension system is more protective towards lower lifetime earnings and early retirement compared to Sweden. In Denmark, replacement rates for low-income workers are comparatively high. Hence, the public pension has a strong redistributive element. This is less the case in Sweden where replacement rates are more similar across the income distribution (OECD 2013).

Both countries provide basic pensions which are comparable with regard to the total amount. However, the full amount of the basic pension in Denmark is only reduced if earnings exceed 75% of average earnings. In Sweden, the basic pension (Swedish: garantipension) is only available as income-tested top up pension for those who earn less than 35% of average earnings. Furthermore, whereas pension withdrawal is possible now already at the age of 61 years in Sweden, the basic pension can still only be withdrawn at age 65 years. Hence, early retirement in Sweden is financially difficult when relying on the basic pension. Eighty-eight per cent of Danish pensioners receive the basic pension and 42% of Swedish pensioners (OECD 2013). A study by Möhring (2015) shows that the impact of career histories on women’s pension income is less strong in pension systems with basic pensions. Following this notion, the generosity of the Danish basic pensions may prevent women from compensating lower career attachment.

In addition to broad basic pensions in Denmark, a rather generous early retirement scheme (Danish: efterløn) was still available at age 60 years after the reforms until 2014 when the entry age started to increase gradually (Beskæftigelsesministeriet 2016). The “transitional benefit program” (TBP, Danish: Overgangsydelse) granted even earlier benefits starting as early as 50 years, but it was only available in the early 1990s (introduced in 1992 and abolished in 1996) (Jensen 2004). In Sweden, disability pensions are a major pathway to early retirement, especially for lower-educated workers and women. However, throughout the reforms, eligibility for this kind of pension was restricted. The so-called 58.3 pensions described the situation until 1991 when a worker who became unemployed at the age of 58 years and 3 months could go into early retirement by a combination of unemployment benefits and disability pensions (Delsen 1996; König and Sjögren Lindquist 2016). Until 1997, disability pensions could still be granted on a combination of medical and labour market grounds. Afterwards, labour market reasons were abolished and medical reasons became stricter (Hamblin 2013). Hence, workers who could still leave early with this kind of pension until the late 1990s have to remain longer at their jobs or accept more severe pension cuts after reforms. Concurrently, occupational pensions gained importance which may pose a threat to low-income workers. Data from the Social Policy Indicators Database (SPIN 2015) show that replacement rates for state pensions dropped from 69.6 in 1990 to 43.4% in 2010 in Sweden for a standard production worker (own estimations). Hence, occupational and private pensions obtained greater importance. This strong drop cannot be observed in many other countries, including Denmark where replacement rates dropped only about 6 percentage points from 54.9% in 1990.

A report on the reformed pension systems in Europe (Natali and Stamati 2013) argues that career interruptions due to childcare and shorter unemployment periods (up to three years) are comparatively well protected in Sweden. Pension entitlements in Sweden are also linked to sickness, unemployment or parental benefits (Anxo et al. 2012). However, the reforms changed the calculation of pension benefits in the new system, starting in 1994. While it was previously calculated on the basis of 15 years of highest earnings, it is now calculated on entire lifetime earnings. Hence, incentives are given for retiring later and interrupted careers are penalized. A Swedish study (Sjögren Lindquist 2011) suggested that after controlling for income at age 60 years, Swedish women retire later in order to accumulate more benefits to make up for career interruptions. Furthermore, according to the report “Part-Time Work in the Nordic Region”, the pension system in Sweden is more actuarial compared to Denmark. Pension losses due to part-time work are relatively small in Denmark but higher in Sweden (Lanninger and Sundstroem 2014). Looking at old age poverty risk rates in nine European countries, the study by Ebbinghaus and Neugschwender (2011) finds the largest gender differences in Sweden, where the risk is twice as high for women. Therefore, I expect the compensation hypothesis to play a role for Swedish women, especially regarding part-time work.

#### **H2b**

The compensation hypothesis is less relevant in Denmark compared to Sweden.

The changes in the pension system may increase the financial need to continue working. Hence, among retirees who are affected by these changes, the compensation hypothesis may be more relevant. All in all, this situation should be more severe in Sweden, where the reforms were more drastic compared to Denmark.

#### **H2c**

The compensation hypothesis is more relevant for later cohorts, especially in Sweden.

## Data and method

The third wave of SHARE, called SHARELIFE (Börsch-Supan 2016), was used for the analysis. It included life history interviews with detailed information on job histories (Antonova et al. 2014). Respondents were age 50 years or older, so all respondents have a retrospective history of at least 50 years. Data for SHARELIFE were collected in 2008/2009 and provide a variety of work and career variables as well as family characteristics, offering a unique setup for researching retirement across countries.

To investigate the timing of retirement, survival analysis is needed to account for right-censoring of events. I use log-logistic regressions on the timing of the final labour market exit, including a censoring indicator for those who did not experience the event at the time of observation. Compared to, e.g. Cox regressions, this method has the clear advantage that comparable marginal effects can be shown which are easy to interpret as years left earlier or later than the predicted median exit age. Furthermore, this comparability is important since the analysis was separated by country, gender and cohort. As a robustness check, Cox regressions were performed which confirmed the directions of the effects, however, with lower statistical significance.

The cohort differentiates those who stopped working before the year 2000 and those who stopped in 2000 or later since most reforms were implemented by the late 1990s. Given the developments and institutional changes between these cohorts, the arguments of the compensation hypothesis can be expected to apply only to those who were affected by the reforms. By differentiating between those exit cohorts, most individuals from the later cohort were affected by most reforms whereas most individuals from the earlier cohort were not. A later cut-off would increase the chance of including individuals in the earlier exit cohorts, despite being affected by the reforms. Another option would be to separate by birth cohort since regulations of the reforms are often related to the year of birth. However, in this case, persons who retired for example in the mid-1990 s could not have been affected by the new reforms, irrespective of their age. It has to be acknowledged that this is a rather rough measure which oversamples those who retire at higher ages in the later retirement cohort. Nevertheless, it still provides valuable insights on different mechanisms across cohorts.

The respondents’ age at their last year of work was used as duration of the dependent variable. Years of unemployment before retirement were treated as being retired. Compared to the age of retirement, this has the advantage that the analysis is irrespective of periods of unemployment before retirement which increase retirement age, but not old age employment. Since the last year of work is typically the year of retirement, this term will be used throughout this article. The failure event was constructed when a person stopped working after the age of 50 years and never returned to the labour market during the time of observation in SHARELIFE. In case a person was observed before the age of 60 years, the reason for leaving the last job had to be retirement. The underlying argument behind this decision is that individuals in their 50s are more likely to return to the labour marked when they were non-employed for some time, which is more rarely the case for those in their 60s. Work status was derived from a self-reported working spell in the life history calendar.

Approximately 15% of Danish women, 10% of Swedish women, and around 5% of men in both countries stopped working before the age of 50 years and were excluded from the analysis. Furthermore, the sample was restricted to individuals who exited the labour market in 1980 or later and who started working no later than age 25 years. This age restriction is necessary since a later labour market entrance is often due to higher education and shortens the duration of total working years. Hence, without this restriction, shorter working years may rather describe a specific educational effect. For the regression analysis, the sample was restricted to individuals who stopped working between the age of 50 and 75 years. Less than 4% leave the labour market after the age of 75 years, according to the SHARELIFE data. Due to the high selectivity of this group and the risk of a coding mistake, those are excluded in the analysis. These restrictions reduce the total sample of 3971 participants to 3286 individuals. The final analysis sample consists of 2958 individuals, who have no missing data on any of the used variables.

Work characteristics of the main job are included as control variables since they are frequently shown to influence retirement timing (Carr et al. 2015; Dal Bianco et al. 2015) and strenuous work is particularly related to the uptake of early retirement (“efterløn”) in Denmark (Lund and Villadsen 2005). Strenuous work was covered by an additive index on job strain which contained the items “work was physically demanding”, “work was uncomfortable”, “work had heavy time pressure”, “work was emotionally demanding” and “work involved conflicts”, each ranked on a four point scale. The index was reversed and ranged from 1 to 16 with higher values indicating higher strain. Self-employment as last job is included with a dummy variable. The educational level was included in three categories, derived from the ISCED-97 categories. The categories 0, 1 and 2 were coded into “low education”, categories 3 and 4 into “medium education” and 5 and 6 into “high education”. Regarding family characteristics, I included the presence of a partner at the time of the labour market exit as dummy variable. Last, as a proxy for health at the time of last labour market exit, leaving the last job due to disability or ill health was included.

To differentiate between interrupted and continuous careers, the total working years between the age of 25 and 49 years were included. Only the career before the possible failure event (50 years or later) was observed to avoid capturing early retirement as part of shorter careers and thereby preclude endogeneity problems. As part-time periods were rare among men, the duration of part-time working years from 25 up to the age of 49 years was only included for women. Since shorter part-time periods cannot be expected to have a strong impact on pension benefits, it was included as a dummy variable, differentiating between those who had at least 10 years of part-time work and those who had less or no part-time work (Table 1).

**Table 1:** Sample description

	Denmark		Sweden	
	Men	Women	Men	Women
	Mean (SD)	Mean (SD)	Mean (SD)	Mean (SD)
Work strain	7.07 (2.52)	7.32 (2.71)	7.50 (2.86)	7.56 (2.88)
High education	31.8%	39.5%	31.8%	36.9%
Low education	31.4%	27.7%	37.1%	27.7%
Self-employment	14.9%	8.0%	15.2%	5.1%
Disability	9.6%	11.0%	9.9%	18.3%
Min 10 years part-time		25.1%		28.2%
Work years (25–49 years)	24.51 (1.94)	21.93 (5.01)	24.59 (1.54)	21.35 (5.07)
Partner	83.2%	78.7%	88.0%	79.4%
*N*	734	805	659	760

SD in parentheses

## Results

**Fig. 2:**
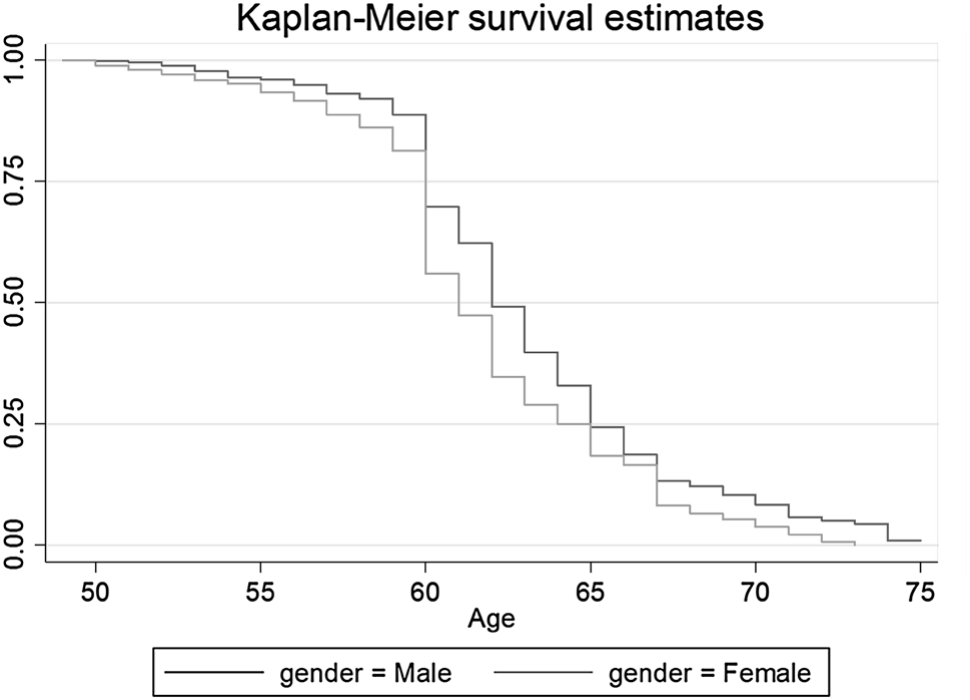
Survival estimates by gender DENMARK

**Fig. 3:**
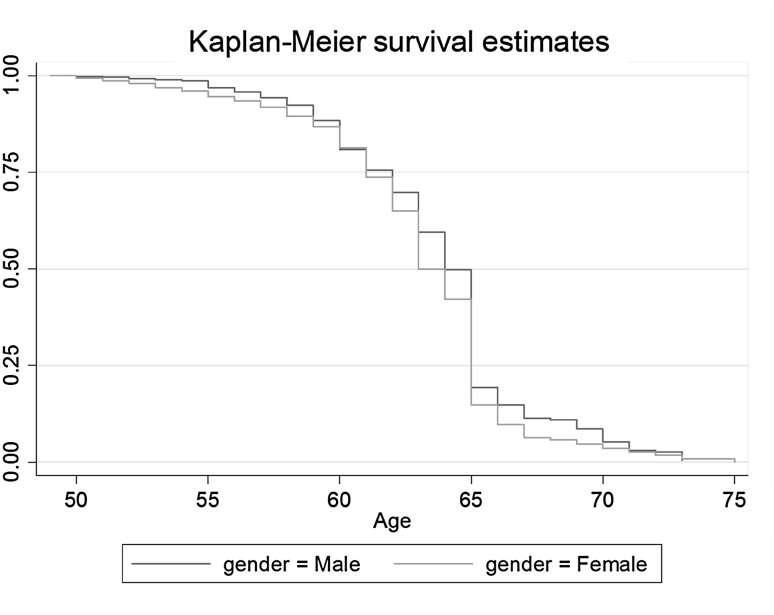
Survival estimates by gender SWEDEN

The descriptive results indicate Kaplan–Meier survival estimates, separated by gender (Figs. 2, 3).

The graphs picture survival estimates for men and women who left the labour market between the age of 50 and 75 years. In Denmark, among those who worked at the age of 50 years and have uncensored observations, around 55% of women and 70% of men are still active after age 60. In Sweden, no gender gap can be found at age 60 years, when 80% of both men and women are still working. Hence, it appears to be the case that Danish women leave earlier whereas Swedish women remain in the labour market almost as long as men. The largest gender gap in Sweden can be observed at age 63 years. Fifty per cent of women had left the labour market by that age while 50% of men remained until the age of 65. The underlying reasons for these gender gaps shall be investigated in the next section (Tables 2, 3).

**Table 2:** Marginal effects after log-logistic regression on labour market exit age (women)

Year retired	Denmark		Sweden	
	Before 2000	2000 or later	Before 2000	2000 or later
Work strain	−0.092	−.270**	−0.211*	−0.032
High education (*d*)	1.517*	0.055	0.395	1.372***
Low education (*d*)	1.883**	0.350	−0.453	0.855*
Self-employment (*d*)	0.067	1.037	3.831*	4.068***
Disability (*d*)	−4.208***	−4.183***	−3.062***	−1.328**
Min 10 years part-time (*d*)	−1.755**	−0.671	0.088	0.664^+^
Work years (25–49 years)	−0.029	0.004	−0.027	−0.035
Partner (*d*)	0.746	−0.879	0.735	−0.700
Predicted median age	60.14	62.99	61.57	64.36
*N* obs	2504	5191	2623	6388
Subjects	238	567	221	539

(*d*) d*y*/d*x* is for discrete change of dummy variable from 0 to 1

^+^ *p* < 0.10; * *p* < 0.05; ** *p* < 0.01; *** *p* < 0.001

**Table 3:** Marginal effects after log-logistic regression on labour market exit age (men)

Year retired	Denmark		Sweden	
	Before 2000	2000 or later	Before 2000	2000 or later
Work strain	−0.137	−0.438***	−0.021	−0.115
High education (*d*)	0.069	0.380	1.096	−0.034
Low education (*d*)	0.922	0.469	0.879	0.081
Self-employment (*d*)	1.830*	1.552*	0.795	2.675***
Disability (*d*)	−3.812***	−2.015**	−2.715***	−2.634**
Work years (25–49 years)	−0.122	−0.032	−0.397*	−0.305^+^
Partner (*d*)	1.916^+^	0.905	0.336	0.098
Predicted median age	61.71	63.79	62.28	65.22
*N* obs	2251	5812	2800	5598
Subjects	182	552	217	442

(*d*) d*y*/d*x* is for discrete change of dummy variable from 0 to 1

^+^ *p* < 0.10; * *p* < 0.05; ** *p* < 0.01; *** *p* < 0.001

Women’s predicted median age of labour market exit is lower than men’s in both countries. Gender gaps are rather low in Sweden with no clear change over time. In Denmark, the gap is lower for those who retired after 1999. Hence, women are “catching up” according to the predicted median ages. The regression results shed some more light on the reasons for gendered retirement timing.

There is country-specific evidence for the compensation *and* the status maintenance hypotheses. Danish women seem to leave earlier when they had been working part-time for more than 10 years, thereby following the status maintenance hypothesis (H1).

In contrast, Sweden rather provides evidence for the compensation hypothesis. Even though evidence is not very strong and some effects do not show high statistical significance, the compensation argument is manifested in several results (H2a and H2b). First of all, Swedish women leave around half a year (Table 3: 0.664 years) later in the younger cohort (year 2000 or later) when they had been working part-time throughout a longer period of their life compared to those with shorter or no part-time periods. The effect for lower-educated women provides additional evidence for the compensation hypothesis. Lower-educated women leave later than those with medium level education in the later cohort which may be interpreted as a need to stay longer at work to accumulate higher pension benefits. Furthermore, Swedish women with high job strain left the labour market significantly earlier before the reforms which is not the case today. They may need to stay longer despite arduous working conditions. Lastly, looking at those who stopped working due to disability, Swedish women in earlier cohorts left around 3 years earlier (Table 3: −3.062 years) than the predicted median age. Hence, they left around the age of 58.5 years. After the reforms, they still leave significantly earlier (Table 3: −1.328 years), but at a much higher age of 63 years which may be related to the severe restrictions in access to disability pensions.

All “compensation effects” are stronger in the more recent retirement cohort, supporting hypothesis H2c. Compensating for lower earnings seems to be more relevant in Sweden, while no such evidence can be found in Denmark where the particularities of the pension system are rather protective for low earners. Thus, Danish women with lower attachment (can) leave earlier, following the status maintenance hypothesis. However, this effect decreases in the later retirement cohort and slightly misses significance. While Danish women in the earlier retirement cohort left 1.755 years earlier than the predicted median age (60.14 years) when they worked part-time for longer than 10 years, they only leave somewhat more than half a year earlier in the later cohort (Table 3: −0.671 years). The overall picture in both countries describes the emerging importance of the compensation hypothesis and the decreasing importance of the status maintenance hypothesis for women.

Comparing those results to men, the pattern of the emerging importance of the compensation hypothesis and the decreasing importance of the status maintenance hypothesis cannot be found. Swedish men, who stopped due to disability, left the labour market around two and a half years earlier in both cohorts (Table 2: −2.715/−2.634 years). Hence, they left around the age of 59.6 years in the earlier cohort and around 62.6 years in the later cohort. The general tendency for longer working lives can be detected among men as well, although to a lesser extent compared to women. Furthermore, the effects of lower education and high work strain, which confirmed the compensation idea for women, cannot be found among men. When it comes to the number of working years, Swedish men tend to leave earlier when they had longer careers during midlife. This effect was slightly stronger in the earlier cohort, again not confirming the suggested rise of the compensation hypothesis. All in all, the compensation hypothesis seems to be stronger in Sweden, among women, and in later cohorts.

Hence, the Danish pension system seems to be more protective and still allows for early exits. Interpreting the results for work strain, this idea is further confirmed. The connection between early retirement/the uptake of “efterløn” and strenuous working conditions (Lund and Villadsen 2005) can clearly be found, especially in the later cohort. While Swedish women currently do not or cannot retire earlier, Danish men and women leave significantly earlier when they endure high work strain.

## Discussion and conclusion

This study provides initial indications for the importance of career histories in a changing institutional context. However, in particular, the missing effect for the total number of years worked is surprising given the lower accumulated pension income. There may be several explanations for these weak findings. First, contrasting theoretical notions may be contributory: The status maintenance hypothesis and the compensation hypothesis argue in opposing directions for the same scenario; certain groups may respond differently, possibly depending on health status and individual or household income. A second explanation for insignificant results may be due to sample selection: women with rather low labour market attachment might drop out before age 50 years. Thus, low labour market attachment may have a stronger influence on earlier dropouts but less on retirement age. It has to be acknowledged that the group of women and men who worked until 50 years may be seen as a selective group since up to 15% stopped working before their 50s. This needs to be considered when investigating other late life outcomes such as pension income. Lastly, the particularities of the welfare state may be an explanation for weak findings of the compensation hypothesis since it is comparatively protective towards shorter interruptions in both countries.

### Limitations

The final labour market exit is difficult to capture since retired individuals might return to the labour market. At the moment, the group of those who return to the labour market is limited (Pleau and Shauman 2013), but it should be considered in future research. The dataset does not provide detailed information on health at the point of employment exit, so the reason for leaving the last job due to disability or ill health was used as a proxy. Furthermore, even though there is information on (pension) income in the dataset, there are many missings on this variable which strongly reduced sub-sample sizes and does not allow for complex analysis. Future studies should investigate the influence of career histories and job characteristics under the control of individual health status and pension income. Nevertheless, this article was able to shed more light on the financial situation on a macro level by comparing different pension systems and changes due to reforms.

### Contributions

The current study provided further information to the partly contradictory results for the relationship between lifetime work participation and retirement timing. While some previous studies found indications for a status maintenance hypothesis in cases where long, continuous careers led to later exits, other studies supported the compensation argument where interrupted careers led to later exits. This paper highlights the importance of the particularities of pension systems with regard to the compensation hypothesis and supports previous findings on pension income (Möhring 2015). Denmark offers comparatively generous early retirement schemes and has high replacement rates for low earners. Accordingly, the compensation hypothesis was found to be less relevant in Denmark. In fact, women in this country tended to follow the status maintenance principle. Still, a shift may be observed and the status maintenance hypothesis is less relevant for the later cohort. This is in line with findings of the compensation effect which was found for women in Sweden. There seems to be a tendency for a weakening of the status maintenance and a strengthening of the compensation hypothesis in the two Scandinavian countries. In particular in Sweden, this might be attributed to the drastic changes in pension systems where early retirement was financially penalized. The increase in employment rates of older workers in Sweden apparently came along with an increase in financial need-driven later retirement for women. This is in line with previous results from Swiss women (Madero-Cabib et al. 2016). Even though the effects for career histories are not strong and need to be further investigated with different datasets, the underlying argument is corroborated by other effects of work strain, disability as reason for labour market exits and education. In Sweden, lower-educated women and those with high work strain tend to exit the labour market later in the younger cohort. One explanation for this finding could be the financial need to work longer despite (potentially) strenuous working conditions or poor health. This finding has important implications for life after retirement and research on ageing. Today, there seems to be a higher risk of certain vulnerable labour market groups to be faced with a trade-off in terms of retirement decisions. Some may have to work longer to increase their pension income. This can even be necessary if they have poor health or unfavourable working conditions which might affect their later wellbeing negatively. Those who retire earlier are accepting lower pension benefits, facing a higher risk of old age poverty. This may be necessary if their workplace or health does not allow for prolonged working lives. That can be of particular importance for all labour market groups who had low lifetime earnings.

Male careers are more continuous and part-time work hardly exists. As a possible result, previous careers did not play a strong role for men in both countries, and they do not follow the same pattern. Today, it seems that the marketization and privatization of pensions is affecting women to a stronger degree. However, due to the continuing flexibilization of the labour market (Hinrichs and Jessoula 2012), it can be assumed that even men could be affected negatively by the reformed pension system in the future.
